# Microvascular and lymphatic dysfunction in HFpEF and its associated comorbidities

**DOI:** 10.1007/s00395-020-0798-y

**Published:** 2020-05-25

**Authors:** Ilona Cuijpers, Steven J. Simmonds, Marc van Bilsen, Elżbieta Czarnowska, Arantxa González Miqueo, Stephane Heymans, Annika R. Kuhn, Paul Mulder, Anna Ratajska, Elizabeth A. V. Jones, Ebba Brakenhielm

**Affiliations:** 1 Center for Molecular and Vascular Biology, KU Leuven, Herestraat 49, bus 911, 3000 Leuven, Belgium; 2 Department of Cardiology, Cardiovascular Research Institute Maastricht (CARIM), Maastricht University, Universiteitssingel 50, 6229 ER Maastricht, The Netherlands; 3 Department of Physiology, Cardiovascular Research Institute Maastricht (CARIM), Maastricht University, Universiteitssingel 50, 6229 ER Maastricht, The Netherlands; 4 Department of Pathology, The Children’s Memorial Health Institute, Aleja Dzieci Polskich 20, 04-730 Warsaw, Poland; 5 Program of Cardiovascular Disease, Centro de Investigación Médica Aplicada (CIMA), Universidad de Navarra, IdiSNA, Avda. Pío XII 55, 31008 Pamplona, Spain; 6 Centro de Investigación Biomédica en Red Enfermedades Cardiovasculares (CIBERCV), Instituto de Salud Carlos III, Av. Monforte de Lemos 3-5, 28029 Madrid, Spain; 7 Netherlands Heart Institute, Holland Heart House, Moreelsepark 1, 3511 Utrecht, The Netherlands; 8 Institut National de la Santé et de la Recherche Médicale (Inserm) UMR1096, Faculty of Medicine and Pharmacy, Normandy University, 22 Boulevard Gambetta, 76183 Rouen, France; 9 Department of Pathology, Medical University of Warsaw, Chalubínskiego 5, 02-004 Warsaw, Poland

**Keywords:** Heart failure with preserved ejection fraction, Coronary microvascular dysfunction, Cardiac lymphatic dysfunction, Inflammation, Myocardial fibrosis, Cardiac metabolism

## Abstract

Heart failure with preserved ejection fraction (HFpEF) is a complex heterogeneous disease for which our pathophysiological understanding is still limited and specific prevention and treatment strategies are lacking. HFpEF is characterised by diastolic dysfunction and cardiac remodelling (fibrosis, inflammation, and hypertrophy). Recently, microvascular dysfunction and chronic low-grade inflammation have been proposed to participate in HFpEF development. Furthermore, several recent studies demonstrated the occurrence of generalized lymphatic dysfunction in experimental models of risk factors for HFpEF, including obesity, hypercholesterolaemia, type 2 diabetes mellitus (T2DM), hypertension, and aging. Here, we review the evidence for a combined role of coronary (micro)vascular dysfunction and lymphatic vessel alterations in mediating key pathological steps in HFpEF, including reduced cardiac perfusion, chronic low-grade inflammation, and myocardial oedema, and their impact on cardiac metabolic alterations (oxygen and nutrient supply/demand imbalance), fibrosis, and cardiomyocyte stiffness. We focus primarily on HFpEF caused by metabolic risk factors, such as obesity, T2DM, hypertension, and aging.

## Introduction

More than half of the patients with heart failure (HF), notably women, suffer from HF with preserved ejection fraction (HFpEF; EF > 50%), a complex cardiovascular syndrome characterised by diastolic dysfunction and cardiac stiffening, fibrosis, inflammation, and hypertrophy. As a consequence of population aging, as well as the increases in common comorbidities, such as obesity, type 2 diabetes mellitus (T2DM), and hypertension, the prevalence of HFpEF is rising [115]. Alarmingly, there are no specific treatments for HFpEF, likely due to incomplete pathophysiological understanding of the underlying mechanisms, patient population heterogeneity, and inadequate diagnosis [110]. In this review, we summarize current evidence of coronary microvascular dysfunction linked to chronic low-grade inflammation, oxidative stress, and microvascular wall barrier dysfunction in HFpEF. Furthermore, we discuss the potential role of lymphatic dysfunction in HFpEF development. We review the role of microvascular and lymphatic dysfunction in mediating reduced left ventricular (LV) compliance (both cardiomyocyte stiffness and cardiac fibrosis) and cardiac metabolic changes occurring during HFpEF development. In each case, we will focus on what is known about these topics in both HFpEF and the commonly associated comorbidities. Finally, we highlight possible therapeutic approaches for HFpEF.

## Coronary microvascular dysfunction in HFpEF

**Fig. 1:**
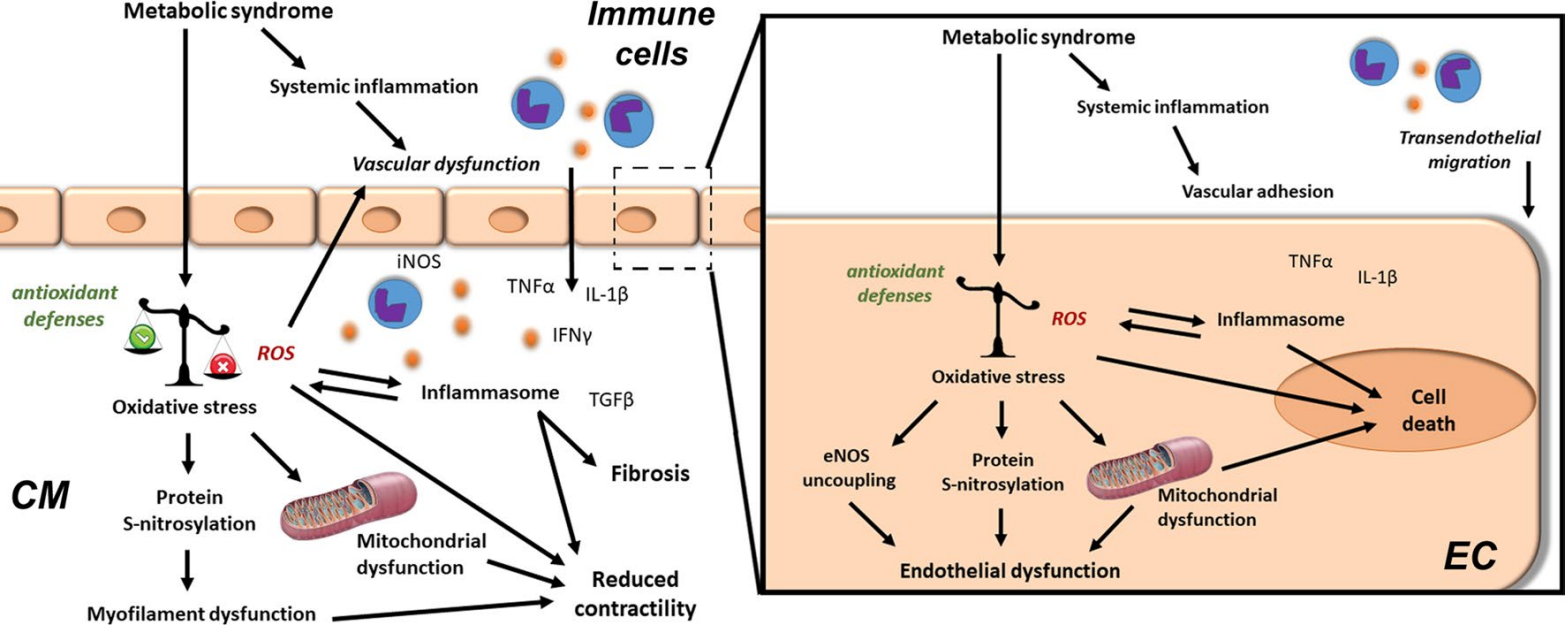
Cardiac and vascular oxidative stress and chronic low-grade inflammation in HFpEF. The metabolic syndrome (obesity, type 2 diabetes mellitus, hypercholesterolaemia, and hypertension) induces chronic systemic low-grade inflammation, as well as direct deleterious effects in the heart (left) and in its coronary endothelium (right). Chronic cardiac low-grade inflammation develops due to increased transmigration of immune cells across activated endothelial cells (EC). Furthermore, endothelial and cardiomyocyte (CM) oxidative stress result from an imbalance between antioxidant defences and reactive oxygen species (ROS) production. Immune mediators, e.g. tumor necrosis factor (TNF)-α, interferon (IFN)-γ, and interleukin 1 (IL)-1β, further increase ROS production. Prolonged ROS-mediated inflammasome activation and the resultant increased transforming growth factor (TGF)-β levels alter the expression of pro-fibrotic genes, contributing to cardiac fibrosis. Furthermore, severe oxidative stress causes lipid, protein, and DNA alterations, leading to mitochondrial dysfunction ultimately resulting in poor cardiomyocyte ATP production, calcium handling, and contractility. In addition, ROS-induced protein modifications (e.g. *S*-nitrosylation) lead to sarcomeric myofilament dysfunction and reduced endothelial nitric oxide synthase (eNOS)-mediated nitric oxide (NO) production. In parallel, oxidative stress leads to eNOS uncoupling, contributing to poor flow-mediated vasodilation and cardiac perfusion. This further aggravates the cardiomyocyte energy supply-demand imbalance. Furthermore, increased myocardial activation of inducible nitric oxide synthase (iNOS) leads to increased nitrosative stress. Finally, persistent vascular pro-inflammatory activation and oxidative stress may induce endothelial cell death, contributing to vascular rarefaction and reduced cardiac perfusion

Vascular endothelial cells constitute the majority of the non-cardiomyocyte population in the healthy heart, therefore cardiac endothelial structural and/or functional abnormalities have major impacts on cardiac health. Coronary macrovascular dysfunction has been scarcely investigated in HFpEF. However, the current *microvascular paradigm* proposes endothelial dysfunction as the central mediator connecting chronic systemic low-grade inflammation with myocardial dysfunction and remodelling in HFpEF (Fig. 1) [94]. In this model, metabolic syndrome (MetS)-related comorbidities, such as obesity, T2DM, and hypertension, trigger chronic systemic low-grade inflammation, characterised by elevated levels of circulating immune cells and pro-inflammatory cytokines and upregulation of endothelial adhesion molecules, such as intercellular and vascular cellular adhesion molecule-1 (ICAM-1 and VCAM-1), and corresponding ligands on circulating leucocytes. The resultant increased myocardial infiltration of leucocytes, especially monocytes, elevates cardiac transforming growth factor beta (TGFβ) levels, thereby inducing cardiac fibrosis. Furthermore, the systemic pro-inflammatory state causes coronary microvascular endothelial cells to produce excessive reactive oxygen species (ROS), contributing to cardiac oxidative stress resulting in oxidation of nitric oxide (NO). Consequently, the reduced NO bioavailability leads to impaired nitric oxide/cyclic guanosine monophosphate/protein kinase G (NO/cGMP/PKG) signalling, causing vascular endothelial dysfunction and cardiomyocyte hypertrophy and stiffening. Decreased NO bioavailability, increased leucocyte infiltration, oxidative stress, and/or neurohormonal activation trigger coronary microvascular endothelial dysfunction and reduced flow-mediated dilatation, which adversely impact cardiac perfusion, as observed in most HFpEF comorbidities (Table 1) [34, 94].

**Table 1:** Mechanistic unravelling in HFpEF and its associated comorbidities

	T2DM	Aging	Hypertension	Obesity	HFpEF
*Systemic alterations*					
Inflammation	**↑** [98]	**↑** [15]	**↑** [116]	**↑** [51]	**↑** [24, 80, 111]
Glycocalyx remodelling	**↓** [86]	**↓** [75]	**↓** [50]	*↓* [31]	N.D
Vascular hyperpermeability	**↑** [79]	*↑* [11]	**↑** [128]	*↑* [55]	N.D
Lymphatic dysfunction	↑ [69, 87]	**↑** [25]	↑ [134, 135]	*↑* [134, 135]	N.D
*Cardiac alterations*					
Microvascular density	**↓** [7]	**↓** [85]	**↓** [3, 88]	**↓** [16]	**↓** [84]
Oxidative stress	**↑** [53]	**↑** [91]	*↑* [17]	**↑** [37]	**↑** [35]
Fibrosis	**↑** [103]	**↑** [113]	**↑** [99]	↑ [90]	**↑** [58, 84, 136]
Metabolic switch to FA beta-oxidation	**↑** [95]	**↓** [60]	**↓** [28]	**↑** [43, 66]	N.D

Evidence from clinical studies given in bold, while proof from experimental studies is indicated in italic

*N.D* not determined, *FA* fatty acid

Coronary microvascular dysfunction may be determined by *endothelium-dependent and/or -independent mechanisms*. Endothelium-dependent dysfunction develops due to an imbalance between endothelium-derived relaxing factors (e.g. NO) and constrictors (e.g. endothelin 1) [127]. In HFpEF patients, plasma levels of NO metabolites were lower compared to HFrEF subjects, indicating a reduced NO bioavailability [19]. In contrast, in moderate regional ischemia, the NO production is increased due to elevated inducible nitric oxide synthase (iNOS) expression, thereby reducing endothelium-dependent vasodilation [46]. Furthermore, HFpEF patients showed increased levels of endothelin-1, a predictor of 1-year HF hospitalisation associated with long-term mortality [21]. Endothelium-dependent coronary microvascular dysfunction was present in 29% of the HFpEF patients, notably those presenting with a greater burden of T2DM and lower high-density lipoprotein (HDL) levels [133]. On the other hand, endothelium-independent dysfunction is the result of changes in vascular tone mediated by an imbalance between vasoconstrictors (e.g. angiotensin II) and vasodilators (e.g. adenosine) acting on vascular smooth muscle cells. A recent study showed that 33% of the HFpEF patients, mostly older, hypertensive subjects, displayed endothelium-independent dysfunction, as reflected by reduced coronary flow reserve (CFR) [133]. Interestingly, this endothelium-independent microvascular dysfunction was associated with a worsened diastolic function and increased mortality [133]. In experimental swine models and diabetic patients, metabolic risk factors (e.g., hypercholesterolaemia, T2DM, and chronic kidney disease) reduced CFR by increasing the basal blood flow following perturbations in myocardial efficiency [9, 41, 96, 121]. Of note, increased basal myocardial blood flow correlated with diastolic dysfunction in female T2DM patients, while CFR did not [41]. As such, basal myocardial blood flow could represent a superior marker of coronary microvascular dysfunction in certain pathological settings [8]. Nevertheless, while administration of the smooth muscle cell relaxants, such as sodium nitroprusside, improves endothelium-independent vasodilation in HFrEF [62], its use in HFpEF is debated [109].

In parallel to these functional vascular alterations, a reduction in myocardial microvascular density, called *microvascular rarefaction*, is observed in HFpEF patients (Table 1) [84]. Capillary rarefaction contributes to insufficient cardiac perfusion by impairing myocardial oxygen delivery in HFpEF patients [123]. Rarefaction of resistance vessels, including small arteries and arterioles, increases microvascular coronary resistance, resulting in reduced cardiac perfusion. Interestingly, in a multiple comorbidity swine model, experimental reductions in myocardial blood flow led to increased myocardial oxygen extraction [121]. This increase occurred despite a reduction in coronary capillary density [121]. As such, rarefaction or dysfunction of coronary resistance vessels was proposed to be responsible for the observed impairment of myocardial blood flow and oxygen delivery [121]. Notably, reduced cardiac perfusion leads to local blood supply-demand imbalance and energy metabolite deficiency, causing cardiac metabolic reprogramming and dysfunction. More than 50% of the patients with coronary microvascular dysfunction had an impaired CFR, which was independently associated with a worsened diastolic function and increased hospitalisation for HFpEF [119]. Microvascular rarefaction may precede disease development, as HFpEF-associated comorbidities show microvascular rarefaction (Table 1). For example, microvascular rarefaction is suggested to impede insulin delivery to muscles and adipose tissue, contributing to poor insulin uptake [7]. In addition, in young adults with familial predisposition to hypertension and in patients with borderline or established hypertension, a reduced dermal capillary density has been shown [3, 88]. In obese patients, increased LV filling pressure correlated with lower coronary microvascular density, potentially contributing to impaired cardiac metabolism underlying diastolic dysfunction [16]. Furthermore, increased subepicardial and pericoronary adipose tissue, as observed in obese, T2DM, and elderly patients, correlated with an impaired CFR, microvasculature, and coronary function, leading to deteriorated diastolic function [85]. However, despite accumulating evidence for coronary microvascular dysfunction and rarefaction during HFpEF development, its exact role in disease progression is still unknown.

### Inflammation as a trigger of coronary vascular dysfunction

The most frequent HFpEF-associated comorbidities are all associated with *chronic systemic low-grade inflammation* (Table 1 and Fig. 1) [15, 51, 98, 116]. HFpEF patients showed elevated systemic inflammatory markers, such as acute inflammatory C-reactive protein (CRP), which increased with the number of comorbidities, and raised circulating levels of neutrophils and monocytes [24, 30, 38, 49]. Additionally, in vitro culture of healthy donor monocytes with serum from HFpEF patients promoted alternative anti-inflammatory/pro-fibrotic macrophage differentiation [38].

Both chronic systemic low-grade inflammation and activation of the renin–angiotensin–aldosterone axis (RAAS) lead to endothelial cell activation by upregulating adhesion molecules. Elevated advanced glycation end products (AGEs)/AGE receptor (RAGE) signalling in T2DM stimulates the nuclear factor kappa-B (NFкB) signalling pathway, inducing pro-inflammatory genes and RAGE, forming a vicious cycle of self-renewing pro-inflammatory signals [10]. HFpEF patients showed increased expression of adhesion molecules on the coronary endothelium, together with elevated myocardial infiltration of CD45^+^ leucocytes and CD3^+^ T-lymphocytes [129]. Furthermore, there was a positive correlation between echocardiographic indices of diastolic dysfunction (*E*/*e′*) and splenic activation, suggesting a role of increased splenic myeloid cell oversupply in HFpEF patients [49]. While both systemic and cardiac inflammation have been observed in HFpEF patients, the causal involvement of cardiac inflammation in coronary microvascular dysfunction in HFpEF has never been investigated.

Given the plethora of cardiac detrimental effect triggered by chronic low-grade inflammation, the use of cytokine inhibitors has been extensively investigated in HF patients [77]. The ability to translate this to a drug has not met success and in some cases has even led to worsening of HF and/or death [77]. Nevertheless, IL-1β blockage (Anakinra), for example, improved aerobic exercise capacity in HFpEF patients and is currently investigated in a phase 2 clinical trial (NCT02173548) [126]. Furthermore, the multi-cytokine blocker Pentoxifylline reduced vascular events, systemic inflammation, all-cause mortality, and improved the prognosis in HFrEF patients [18]. In addition to anti-cytokine therapies, lipid-lowering statins have anti-inflammatory effects and are associated with a reduced re-hospitalisation and mortality in HFpEF patients [78]. Despite this, currently no approved effective anti-inflammatory drug has been approved for the treatment or prevention of HFpEF.

### Oxidative stress as a trigger of coronary vascular dysfunction

*Oxidative stress* is induced by increased ROS production and/or reduced antioxidant enzyme levels, leading to both endothelial and cardiac dysfunction (Fig. 1). As cardiomyocytes are rich in mitochondria, they have an elevated baseline ROS production compared to other cell types. Thus, altered mitochondrial function and/or reduced antioxidant enzyme levels lead to cardiac oxidative stress. Of note, risk factors for HFpEF further stimulate ROS production (Table 1) [17, 34, 37, 53, 91]. For example, AGE-RAGE signalling in T2DM induces oxidative stress by directly activating nicotinamide adenine dinucleotide phosphate oxidases (NOX), decreasing the activity of enzymatic antioxidant defences, and indirectly by reducing cellular antioxidant systems [100]. Consequently, chronic systemic low-grade inflammation is proposed as a major trigger, together with oxidative stress and NO dysregulation, for the development of coronary microvascular dysfunction in HFpEF [94].

Within vascular endothelial cells, elevated ROS production triggers canonical NFкB signalling, leading to cytokine production and proteasome and inflammasome activation, which may cause endothelial cell apoptosis and pyroptosis (Fig. 1) [34]. Endothelial oxidative stress accelerates NO degradation by superoxide anion (O_2_^−^)-mediated peroxynitrite (ONOO^−^) formation, thereby promoting protein nitrosylation, resulting in endothelial dysfunction and cell death (Fig. 1) [52]. Increased cardiac levels of hydrogen peroxide (H_2_O_2_) and reactive oxidative metabolites, endothelial nitric oxide synthase (eNOS) uncoupling, and macrophage and endothelial NOX2 expression and reduced NO levels all indicate the presence of myocardial oxidative stress in HFpEF patients (Table 1) [35]. Beyond oxidation, inhibition of NO production could reduce NO bioavailability, by for example AGE-induced elevation of asymmetric dimethyl-l-arginine (ADMA) levels, an eNOS inhibitor, thereby contributing to endothelium-dependent dysfunction associated with a worsened prognosis of HFpEF [5]. Despite the signs of cardiac oxidative stress in HFpEF patients, the causal involvement of cardiac oxidative stress in the development or aggravation of coronary microvascular dysfunction is not well understood.

In HF patients and experimental mouse models, several anti-oxidative stress therapies have been investigated that either (1) inhibit oxidative stress producers, (2) improve endogenous antioxidant capacity, or (3) supplement exogeneous antioxidants. Mitochondria Szeto-Schiller-31 (SS-31; Elamipretide) attenuated cardiac remodelling in hypertensive cardiomyopathy and is currently investigated as a novel therapeutic in phase II trials for HFpEF (NCT02814097). Furthermore, treatment with the mitochondria-targeted antioxidant MitoTEMPO or *N*-acetylcysteine (NAC) prevented diastolic dysfunction in rodent models of diabetes and hypertensive cardiomyopathy [74, 130]. Supplementation with an antioxidant cocktail, containing alpha lipoic acid, vitamin C, and E, also reduced systemic inflammation and improved conduit artery endothelium-dependent vasodilation in HFpEF patients [101]. In summary, therapies targeting oxidative stress could be a potential therapy for HFpEF. However, their underlying mechanism needs to be further elucidated.

### Microvessel wall barrier dysfunction in HFpEF

The microvascular endothelium is a barrier opposing free exchange between blood and tissues, which tightly regulates transport of plasma constituents and immune cells in most organs. Events such as ischaemia cause increased vascular endothelial growth factor (VEGF)-A levels, leading to vascular barrier breakdown, increased extravasation of immune cells, and oedema. In addition to its well-known occurrence in HFrEF, there is accumulating evidence of *microvascular wall barrier dysfunction* in HFpEF-associated comorbidities [11, 55, 79, 128], while its role in the development of coronary microvascular dysfunction has been scarcely addressed in HFpEF (Table 1).

**Fig. 2:**
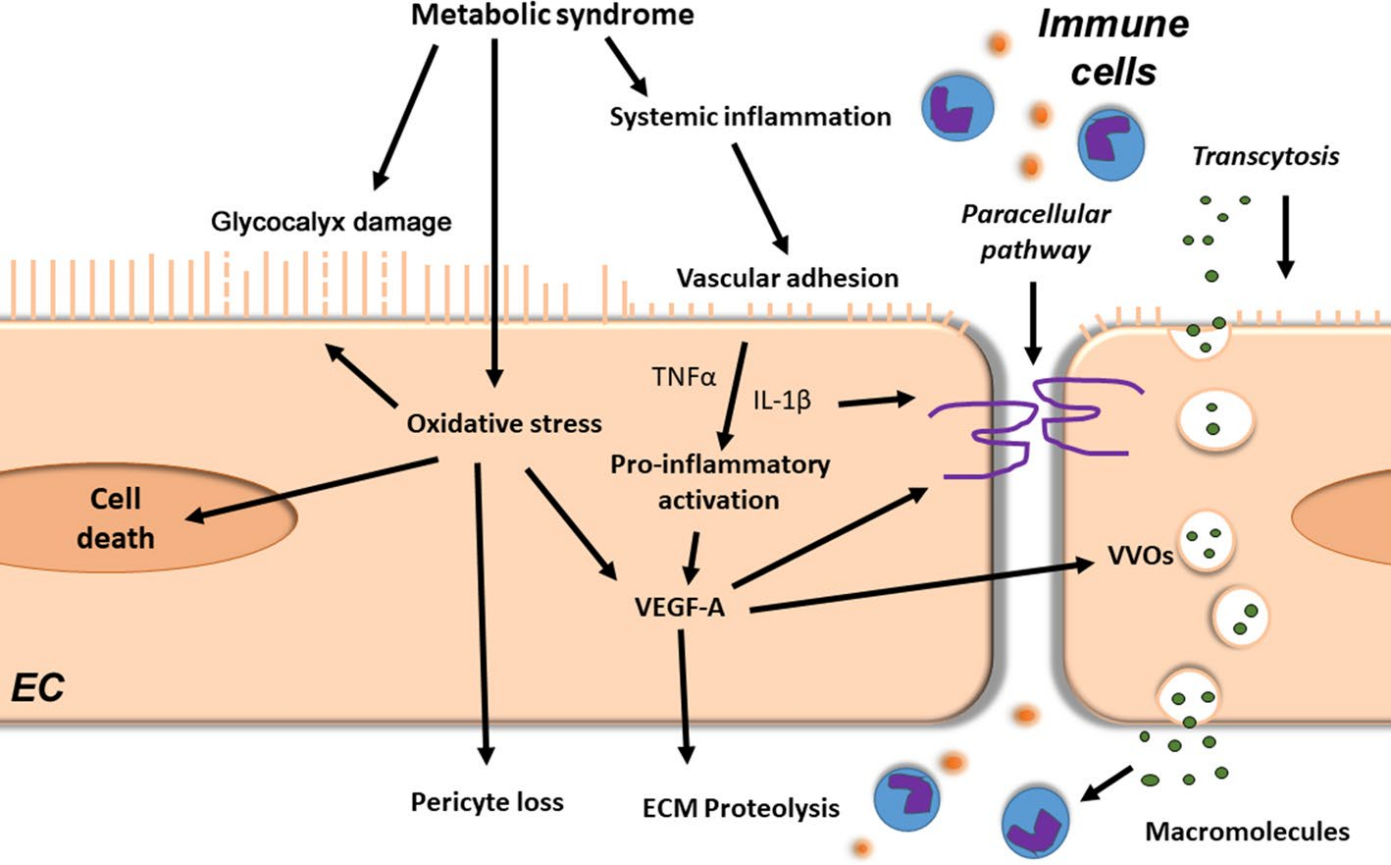
Microvessel wall barrier dysfunction in HFpEF**.** The metabolic syndrome induces via chronic systemic low-grade inflammation deleterious effects in coronary endothelial cells (EC). It leads to the degradation of the endothelial glycocalyx layer, thereby promoting endothelial immune cell adhesion and transmigration. Furthermore, metabolic syndrome-induced cellular oxidative stress may lead to glycocalyx damage and cell death of both endothelial and mural cells. In addition, pro-inflammatory mediators, such as tumor necrosis factor (TNF)-α and interleukin (IL)-1β, together with oxidative stress, increase vascular growth factor (VEGF)-A levels. Increased VEGF-A signalling weakens vascular barriers (e.g. loss of cell–cell junctions), which facilitates paracellular passage of immune cells and trans-vascular transport by transcellular vesiculo-vascular organ (VVO) formation. VEGF-A also stimulates vascular basement membrane remodelling through extracellular matrix (ECM) proteases activation, leading to reduced vascular stability and vascular regression

Vascular barrier function is controlled on several levels by different cell types. For example, the *endothelial glycocalyx* covers the luminal surface of vascular endothelial cells and together with cell–cell junctions serves as a barrier for solute and macromolecule exchanges. It is also a mechanotransducer, which senses endothelial shear stress, attenuates coagulation and leucocyte adhesion to the endothelium, and affects vasoregulatory responses to flow. A damaged glycocalyx induces the production of pro-adhesion mediators, thereby triggering the adherence of neutrophils to the endothelium (Fig. 2) [54]. Thinning of the glycocalyx occurs in several HFpEF-associated comorbidities (Table 1 and Fig. 2) [31, 50, 75, 86]. In HFpEF patients, increased circulating levels of syndecan-1, a glycocalyx shedding biomarker, were associated with endothelial dysfunction and a doubling of plasma syndecan-1 levels increased risk of all-cause mortality and rehospitalisation [120]. Interestingly, exogenous NO administration during reperfusion preserved vascular integrity and attenuated cardiac oedema formation though protection of the glycocalyx in guinea pigs subjected to ischemia/reperfusion injury [14].

*Vascular endothelial hyperpermeability* has been shown to occur in the microvasculature of peripheral tissues of hypertensive or diabetic patients, as well as obese or elderly rodent models [11, 55, 79, 128]. Disruption and remodelling of cell-cell junctions is a major cause of vascular barrier integrity loss and occurs in murine models of diastolic dysfunction and MetS and in aged endothelial cells (Table 1 and Fig. 2) [55, 64, 89, 106]. A number of pro-permeabilizing stimuli, including VEGF-A and inflammatory agents (e.g. histamine and bradykinin), stimulate hyperphosphorylation of vascular endothelial (VE)-cadherin, resulting in the breakdown of junctional contacts [23]. Moreover, the VEGFA-, histamine-, and serotonin-stimulated formation of trans-endothelial channels from coalesced vesicles or vacuoles, called *vesiculo-vacuolar organelles* (VVOs) is another proposed route of transcytosis-mediated extravasation during vascular leakage (Fig. 2). Vascular hyperpermeability by trans- or paracellular pathways leads to increased influx of solutes, macromolecules, and immune cells to the interstitium.

Beyond endothelial cells, *pericytes*, the main mural cell type of microvessels, are crucial for regulating vascular blood flow and microvascular stability. Cardiac pericytes are involved in many processes regulating cardiac homeostasis, such as vascular maturation, supply of trophic substances, fibrosis, and blood flow. HFpEF-diseased ZSF1 rats showed disorganized accumulation of vascular pericytes in subendocardial hyperproliferative (inflamed) foci, while vascular pericyte coverage was reduced compared to controls [122]. Further investigations of microvascular barrier dysfunction in HFpEF are warranted with the aim to develop novel tools for diagnosis or target-specific therapy to limit vascular dysfunction.

## Lymphatic dysfunction in HF

The heart has an extensive lymphatic network that relies on cardiac contractions to propel lymph fluid towards draining cardiac lymph nodes [33]. Cardiac lymphatic drainage is essential for cardiac fluid balance [82]. Insufficient cardiac lymphangiogenesis contributes to myocardial oedema, inflammation, and fibrosis following experimental myocardial infarction in rodents, and it has been shown that cardiac oedema subseqeuntly adversely affects heart function [13, 47]. However, it is unknown whether the same is true for HFpEF. HFpEF patients show increased interstitial water in the lungs, suggesting impaired lymphatic drainage [102]. In addition, decreased left ventricular contractility and relaxation was observed in a canine model of acute lymphatic obstruction, suggesting a role for lymphatic dysfunction in diastolic dysfunction [73]. Furthermore, the generalized oedema that accompanies advanced HFpEF further indicates that lymphatic dysfunction may be involved in HFpEF development and/or progression.

**Fig. 3:**
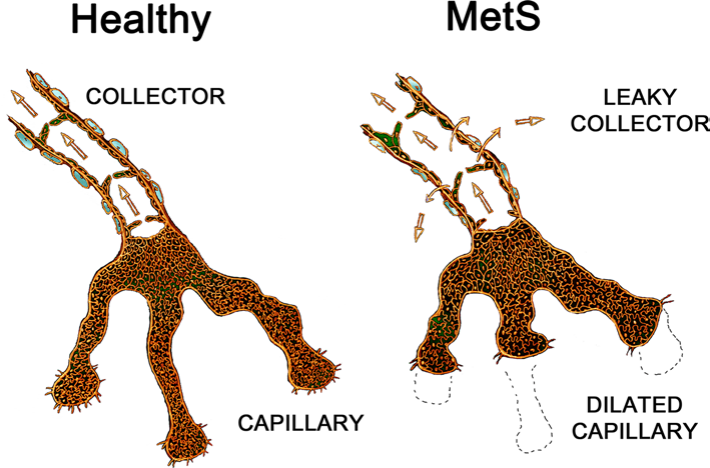
Lymphatic vasculature in the metabolic syndrome. The lymphatic system is composed of highly permeable blunt-ended lymphatic capillaries, which drain into larger collecting lymphatic vessels endowed with valves to prevent backflow and a muscular layer that propulses the lymph towards draining lymph nodes. Experimental models of metabolic syndrome components showed lymphatic dysfunction, including rarefaction, and dilation of initial lymphatic capillaries, but also enlargement, hyperpermeability, and poor contraction of collecting ducts, together resulting in reduced lymphatic transport capacity

*Lymphatic dysfunction and remodelling* has been shown to occur in experimental models of multiple HFpEF-associated comorbidities (Table 1 and Fig. 3) [25, 134, 135]. For example, murine genetic- or diet-induced obesity models showed reduced dermal lymphatic collecting vessel pumping rates, as well as reduced lymphatic capillary density with accumulation of pro-inflammatory cells around lymphatic vessels [69, 87]. These lymphatic-associated immune cells may further reduce collector vessel pumping rates by excessive production of NO via iNOS [69, 87]. Another example is hypercholesterolaemia, which was associated with lymphatic capillary regression, dermal backflow of lymph, dilation of initial lymphatics, and reduced muscular layer coverage in collecting ducts in mice, which all were reversible upon cholesterol-lowering treatment [70]. Rodent models of T2DM showed reduced lymphangiogenesis and lymphatic vascular integrity and function, with insufficient NO bioavailability and poor lymphangiogenesis, contributing to lymphatic permeability and delayed wound healing, respectively [20, 108]. In contrast, T2DM patients had increased dermal lymphatic density and lymphatic endothelial proliferation [42]. Furthermore, canine models of chronic hypertension showed increased myocardial blood capillary permeability accompanied by elevated cardiac lymphatic transport [65]. Interestingly, spontaneously hypertensive rats showed increased VEGF-C-mediated cardiac lymphangiogenesis accompanying LV remodelling [132]. Collectively, these studies indicate an increased demand on the lymphatic system in these HFpEF-associated comorbidities, resulting in maladaptive lymphangiogenesis, as well as hyperpermeability and integrity loss in lymphatics, together leading to lymphatic transport dysfunction. Several studies have shown that immune cells actively participate in lymphangiogenesis and alter lymphatic function [1, 61, 117]. Pro-inflammatory immune cells have been shown to accumulate around lymphatic vessels and reduce lymphatic transport during acute inflammation in models of obesity [69, 87]. Furthermore, while B-cells and neutrophils stimulate lymphangiogenesis, Th2-skewed CD4^+^ helper T-cells have been found to inhibit lymphangiogenesis in tissues such as lymph nodes, skin, and lung [2, 4, 59, 112, 118]. The complexity of immune-lymphatic interactions is further highlighted by the role of the lymphatic system in limiting immune cell residence in tissues [6, 40, 56]. Accordingly, many studies have shown that inhibition of lymphangiogenesis prolongs the inflammatory response and increases oedema at the inflammatory site [6, 40, 56], suggesting that increasing lymphangiogenesis might be a potential novel therapeutic option to reduce chronic inflammation in HFpEF.

*Therapeutic lymphangiogenesis* induced by lymphangiogenic growth factors VEGF-C and -D has shown some potential in terms of treating HFpEF-associated comorbidities. For example, overexpression of VEGF-D increased renal lymphangiogenesis and prevented salt- and NOS inhibition-induced hypertension in mice [72]. Similarly, systemic delivery of VEGF-C lowered blood pressure and preserved cardiac function in salt-sensitive hypertensive rats, while neutralization of both VEGF-C and -D aggravated hypertension and cardiac dysfunction [131]. Finally, therapeutic lymphangiogenesis has been shown to accelerate the resolution of both acute and chronic inflammation in various settings [61]. This shows the potential of therapeutic lymphangiogenesis in treating certain HFpEF-related comorbidities but also highlights lymphatic function and lymphangiogenesis as one of the most underappreciated and unstudied therapeutic opportunities.

## Reduced LV compliance in HFpEF: cardiac fibrosis and cardiomyocyte passive stiffness

Diastolic dysfunction is caused by impaired active relaxation and/or reduced compliance of the ventricle. Both increases in extracellular matrix deposition (fibrosis) and cardiomyocyte passive stiffness (titin modifications) reduce LV compliance, as observed in HFpEF patients [36].

### Increased cardiomyocyte stiffness: titin post-translational modifications and isotype switching

The giant sarcomeric protein titin, a force-transducing bidirectional spring, is the main determinant of cardiomyocyte stiffness. Alternative splicing of titin mRNA creates isoforms with differential stiffnesses: the short, stiffer N2B isoform, and the longer, more compliant N2BA isoform. Extension of the elastic I-band region in both titin isoforms supports myocardial passive relaxation during diastole. Post-translational modifications of titin rapidly alter cardiomyocyte stiffness. In HFpEF patients, hypophosphorylation of N2B and ex vivo administration of PKG are associated with increased LV stiffness [12, 125]. Interestingly, PKG activity is reduced in HFpEF as a result of reduced NO bioavailability due to oxidative stress, leading to impaired NO/cGMP/PKG signalling [94]. This suggests that oxidative stress, NO bioavailability, and PKG play a crucial role in regulating cardiomyocyte stiffness. Furthermore, other protein kinases, such as PKA, PKC, extracellular signal-regulated kinase-2 (ERK2), and Ca^2+^/calmodulin-dependent kinase-II (CAMKII) also modify passive stiffness [36]. HFpEF patients showed increased site-specific titin phosphorylation on PEVK (a region rich in specific amino acids) S_11878_ and reduced phosphorylation on N2B unique sequence (N2Bus) S_4185_, which was associated with increased LV stiffness [136]. In addition to these post-translational modifications of titin, alteration of the N2BA/N2B isoform ratio occurs in pathology [76]. However, HFpEF patients do not consistently show isoform changes, with only some exhibiting increased levels of the stiff N2B isoform [124]. Altogether, increased cardiomyocyte stiffness in HFpEF may be mediated by short term post-translational modifications, which are interconnected with the oxidative state, and by isotype switching in the long term.

### Collagen quantity, type, and cross-linking are altered during HFpEF

Myocardial fibrosis and extracellular matrix accumulation are hallmarks of adverse cardiac remodelling associated with HFpEF and its comorbidities (Table 1) [58, 84, 90, 99, 103, 113, 136]. The quantity, type, and degree of crosslinking of collagen influences tissue stiffness. Increased collagen deposition, switching from more flexible collagen III to stiffer collagen I, and collagen cross-linking is associated with worse diastolic function in HFpEF patients [58, 84, 136]. Furthermore, collagen cross-linking also correlates with increased LV filling pressures and elevated risk of hospitalisation in hypertensive HF patients [39].

Lysyl oxidases (LOXs) are the main enzymes involved in collagen and elastin cross-linking during cardiac remodelling [105]. Upregulation of LOX expression and collagen cross-linking is associated with impaired diastolic tissue Doppler parameters (e.g. *E*/*E′*) in HFpEF patients [58]. Furthermore, AGE-mediated cross-linking of collagen was also observed in aged and T2DM patients, resulting in reduced vascular elasticity and myocardial flexibility, contributing to vascular and myocardial stiffness, and ultimately diastolic dysfunction [29]. Cardiac fibrosis is also associated with cardiac microvascular rarefaction in HFpEF patients, indicating that insufficient cardiac perfusion may cause excessive collagen deposition [84]. Pro-inflammatory mediators (e.g. IL-6, TNFα, CCL2, and TGFβ) participate in fibrosis regulation, and pharmacological prevention of cardiac infiltration of pro-inflammatory monocytes attenuates fibrosis development in murine hypertension [32]. The role of specific cytokines in cardiac fibrosis is, however, debated. Interestingly, cardiac oedema increased collagen production, whereas reduction of cardiac oedema attenuated interstitial cardiac fibrosis in rodent myocardial infarction, suggesting a potential role of oedema and poor lymphatic transport in regulating cardiac interstitial fibrosis [47].

Several anti-fibrotic therapeutics, focused on inhibiting aldosterone signalling with mineralocorticoid receptor antagonists (MRA), have been trialled in patients with in HFpEF [63]. Promisingly, MRA improved cardiac fibrosis markers and diastolic function [92]. Another anti-fibrotic therapy is the AGE-crosslink breaker alagebrium chloride (ALT-711), which improved diastolic function by reducing cardiac stiffness in elderly HFpEF patients (NCT01014572) [71]. While antifibrotic therapies show clinical efficacy in HFrEF patients [93], their effects are more limited in HFpEF patients. One reason for this discrepancy could be the variation in fibrosis pathophysiology found between HFrEF and HFpEF. Indeed, while HFrEF patients present with scar development or “reparative fibrosis” [83], HFpEF patients are much more likely to show interstitial “reactive fibrosis” and perivascular fibrosis [27]. Differences in the mechanisms underlying fibrosis deposition could thus explain why interventions targeting the type of fibrosis that occurs in HFrEF have had limited success in HFpEF patients [57]. Innovative approaches to reduce cardiac stiffening, however, are expected to provide significant benefit in HFpEF.

## Impact of microvascular and lymphatic dysfunction on cardiac metabolism

The healthy heart requires a large, constant supply of energy of which 30% is provided by carbohydrate oxidation (mainly glucose and lactate) and 70% by beta-oxidation of free fatty acids (FFAs) at rest. Cardiac metabolic alterations in HFpEF patients have been poorly investigated and current hypotheses are largely based on metabolic alterations observed in the separate comorbidities (Table 1). For example, in aging and hypertensive LV hypertrophy, there is a decreased reliance on cardiac FFA utilization and beta-oxidation observed [28, 60]. In contrast, insulin resistance and increased circulating triglyceride and FFA levels make cardiac ATP synthesis more dependent on FFA oxidation [43, 66]. Indeed, animal models of obesity and T2DM with diastolic dysfunction show reduced cardiac glucose uptake and increased FFA beta-oxidation [22]. The increased dependence on FFA oxidation in obese and T2DM patients in combination with cardiac microvascular dysfunction and rarefaction might result in a mismatch between oxygen supply and demand [95, 104]. Some studies reported a maintained cardiac creatine phosphate/ATP ratio in T2DM patients, whereas others demonstrated a reduction, suggesting that cardiac energy depletion may occur in T2DM [67, 104]. Impaired cardiac lymphatic transport, as seen in experimental models of HFrEF [47], could cause a build-up of waste products influencing energy metabolism. Intriguingly, whereas angiogenesis depends on active glycolysis, lymphangiogenesis depends on FFA beta-oxidation. It is conceivable that metabolic impairment and increased FFA beta-oxidation in cardiomyocytes during HFpEF may limit substrate availability in cardiac lymphatic endothelial cells, limiting lymphangiogenic responses during cardiac hypertrophy.

Interventions, such as caloric restriction, and insulin-sensitizing or glucose-lowering agents, such as metformin, dipeptidyl peptidase-4 (DPP-4) inhibitors, and glucagon-like peptide-1 (GLP-1) receptor agonists, all improve murine diastolic function [45]. In HFrEF patients, metabolic therapies that limit cardiac FFA beta-oxidation and promote glucose oxidation are promising [45]. Till now only sodium-glucose transport protein 2 (SGLT2) inhibitors (Dapagliflozin) have been proven to be beneficial in HFrEF patients, whereas its therapeutic benefit in HFpEF is currently being investigated [81]. To develop innovative metabolic therapies, further investigations are required to delineate the nature and cause of the alterations in cardiac metabolism in HFpEF and their links to other cardiac changes, such as insufficient perfusion and chronic low-grade inflammation.

## Conclusion

Currently, effective and specific therapies for HFpEF are lacking due to incomplete understanding of disease mechanisms. Advances in this area are hindered in part by the limited number of adequate animal models. Indeed, the disease mechanisms that were discovered in experimental animal studies may be influenced by the short-comings to account for comorbidities, age, gender, and hormonal status of current experimental animal models. Given that HFpEF is such a clinically heterogeneous disease, there may not be one single uniting pathological mechanism driving the disease and, therefore, having multiple pre-clinical animal models that can be compared to human pathophysiology is critical. Due to their similarity to human disease progression, large animal models, such as the multiple comorbidity swine model [114], are especially attractive.

Symptoms of HFpEF are not disease-specific and are often difficult to interpret in patients, leading to inadequate or late diagnosis [26, 44, 107]. Echocardiography alone often fails to demonstrate diastolic dysfunction, resulting in the need for invasive cardiac functional assessment. While multiple algorithms and clinical definitions have been proposed, risk stratification of patients in several HFpEF subgroup has not yet been achieved [97]. As such, there is an urgent need for an improved clinical definition of HFpEF, likely to be driven by the development of novel diagnostic tools, such as cardiac and non-cardiac imaging strategies and early biomarkers for risk stratification.

Nevertheless, it is envisaged that therapies targeting generalized vascular dysfunction, such as (1) anti-inflammatory drugs, (2) antioxidants, (3) anti-vascular permeability drugs (such as glycocalyx, pericyte, and cell–cell junction stabilizers), and/or (4) anti-vascular rarefaction drugs (such as angiogenic therapy) might prevent or treat HFpEF by restoring cardiac perfusion and attenuating cardiac inflammation. The translation of vascular drugs to clinical practice in HFpEF, as well as HFrEF, is still difficult due to the complex and multifactorial pathophysiology of the coronary circulation [48, 68]. In parallel, treatments targeting systemic lymphatic dysfunction, such as VEGF-C or -D lymphangiogenic therapy, might be considered to limit oedema and chronic low-grade inflammation in HFpEF. These vascular therapies are expected to complement other current major therapeutic targets in HFpEF, such as cardiac fibrosis targeting with aldosterone pathway inhibitors, or directly targeting chronic low-grade inflammation and oxidative stress (e.g. pirfenidone). Finally, it remains to be determined if cardiac metabolism in HFpEF patients could benefit from (1) FFA beta-oxidation antagonists (2) glucose uptake agonists, or (3) AMPK agonists.

Notably, as HFpEF is a multifactorial disease, combined personalized medicine targeting cardiac vascular and lymphatic dysfunction, fibrosis, inflammation, and metabolic flux alterations may be needed to limit diastolic dysfunction and improve quality of life.
